# Self-Supervised Stacked Masked Denoising Autoencoder (S^2^MDAE) for Brain MRI Denoising and Feature Learning

**DOI:** 10.3390/s26175388

**Published:** 2026-08-26

**Authors:** Rui Li, Puyu Zhang, Chenglin Wen, Guoxi Sun

**Affiliations:** 1 College of Electronic Information Engineering, Guangdong University of Petrochemical Technology, Maoming 525000, China; lirui@gdupt.edu.cn (R.L.); zhangpy@gdupt.edu.cn (P.Z.); 2 Provincial Key Laboratory of Petrochemical Equipment Fault Diagnosis, Guangdong University of Petrochemical Technology, Maoming 525000, China

**Keywords:** self-supervised learning, masked image modeling, denoising autoencoder, feature learning, brain MRI

## Abstract

Medical-image annotation is costly and limits the use of fully supervised learning. This study proposes the Self-Supervised Stacked Masked Denoising Autoencoder (S^2^MDAE) for four-class brain MRI classification. During pre-training, three convolutional encoder–decoder blocks perform layer-wise masked reconstruction under a 75% mask ratio and Gaussian corruption; the pretrained encoder stack is then fine-tuned for classification. On the primary dataset, S^2^MDAE achieved 87.207% Accuracy, while ablation studies supported the contributions of pre-training, masking, noise injection, and layer-wise reconstruction. Representation analysis further showed increased class separability in deeper blocks after fine-tuning. Without retraining, external evaluation on a duplicate-screened independent dataset achieved 67.254% Accuracy and 66.009% Macro-F1, indicating partial transfer under domain shift. These findings support the task-specific value of the proposed framework while showing that broader clinical generalization requires further validation.

## 1. Introduction

Medical imaging is widely used in clinical diagnosis, and automated analysis can support the identification of brain tumors. Brain magnetic resonance imaging (MRI) classification is one application of this broader area [1]. Although supervised deep learning methods have achieved useful performance, they commonly require substantial amounts of labeled data [2]. Because medical-image annotation requires specialist involvement, the availability of reliable labels can be limited. Self-supervised learning has therefore become a widely studied approach for learning representations from unannotated data [3].

Recent reviews also illustrate the rapid evolution of adjacent magnetic-resonance signal-processing and reconstruction tasks. Zhan et al. survey artificial-intelligence methods for nuclear magnetic resonance spectroscopy, including sparse reconstruction, noise filtering, and artifact suppression, while emphasizing unresolved issues such as signal preservation, quality assessment, data scarcity, and generalization to unseen measurements [4]. Wang et al. review knowledge-driven deep learning for undersampled MR image reconstruction, tracing the transition from supervised to semi-supervised and unsupervised learning and highlighting the importance of incorporating acquisition physics and domain knowledge into reliable reconstruction systems [5]. These reviews concern NMR spectroscopy processing and accelerated MRI reconstruction rather than downstream classification of already reconstructed brain MR images. Nevertheless, they position the present work within the broader movement toward learned reconstruction, corruption-aware modeling, and label-efficient magnetic-resonance analysis. The specific scope of this study is self-supervised representation learning for brain MRI classification, not raw-signal recovery or k-space reconstruction.

The core idea of self-supervised learning is to enable models to learn representations from the data itself by designing pretext tasks, without requiring human-annotated labels [6]. Recent methods such as MAGE (Masked Generative Encoder) [7] and I-JEPA (Image-based Joint-Embedding Predictive Architecture) [8] have demonstrated potential in self-supervised representation learning, though their applicability to medical images remains to be validated. Among pretext tasks in self-supervised learning, Masked Image Modeling (MIM) [9] has shown strong performance in prior studies and draws inspiration from Masked Language Modeling (MLM) in natural language processing. MIM randomly masks portions of the input image and trains the model to predict the masked content, encouraging representations of image structure and contextual information. Denoising Autoencoders (DAEs) [10] reconstruct clean inputs from corrupted observations and can encourage representations that are less sensitive to the corruption patterns used during training. Additionally, contrastive and self-distillation methods such as SimCLR [11], BYOL [12], and MoCo v2 [13] have achieved strong results on natural images. Their performance in MRI can depend on the augmentation design and the semantic consistency of positive views, which motivates task-specific evaluation rather than direct transfer of conclusions from natural-image benchmarks.

Network depth itself is not presented as the distinguishing contribution of this study. Stacked denoising autoencoders have long used cascaded denoising encoders [14], and MAE employs a deep multi-layer encoder [9]. The architectural distinction examined here instead concerns where corruption and reconstruction objectives are introduced and how they are coupled across the network. S^2^MDAE applies independently sampled masks at successive convolutional encoder–decoder blocks, jointly optimizes reconstruction losses at the image and intermediate-feature levels, combines input Gaussian corruption with block-wise masking, and transfers the jointly pre-trained encoder stack to brain MRI classification. These choices are intended to accommodate tumor appearances that vary in scale, shape, and texture [15], but they are described as a task-specific configuration rather than a new primitive architecture.

To address these challenges, this paper develops a Self-Supervised Stacked Masked Denoising Autoencoder (S^2^MDAE) framework that combines masked image modeling, denoising autoencoders, and stacked representation learning [14]. The contribution does not lie in introducing MIM, DAE, or stacked autoencoders as independent concepts. Rather, it lies in their task-specific integration through layer-wise masked denoising reconstruction at successive feature levels, simultaneous masking and Gaussian-noise corruption, and transfer of the resulting hierarchical representations to brain MRI classification. Our method comprises two training stages: a pre-training phase and a fine-tuning phase. During the pre-training phase, the model simultaneously handles two types of input corruption: first, random masking that obscures a large portion (75%) of the input image; second, the addition of Gaussian noise to further increase the difficulty of the reconstruction task [16]. This dual corruption strategy is used as a corruption-based regularizer intended to promote informative representations; its noise tolerance is evaluated separately across multiple Gaussian-noise intensities.

The stacked architecture constitutes a central design choice of this study. Unlike a single-layer autoencoder, it applies masked denoising reconstruction at three successive computational stages and propagates each intermediate representation to the next block. We do not assume a priori that particular blocks exclusively encode image details or semantic concepts. Instead, block-wise activation responses, linear probing, clustering metrics, and t-SNE are used to examine how the representations differ before and after supervised fine-tuning. After pre-training completion, we utilize the learned feature representations as initialization for fine-tuning on downstream brain tumor classification tasks [17].

The methodological contribution of our approach is the coordinated integration and empirical validation of these established components, rather than the introduction of a new primitive learning mechanism. Specifically, the stacked implementation applies masked denoising reconstruction at multiple feature levels to capture representations relevant to medical images. The experiments evaluate the integrated framework on brain MRI classification, while the ablation studies quantify the contribution of each component within the tested configuration. The results provide a reference point for further evaluation of related self-supervised designs on broader medical imaging datasets. Self-supervised representation learning remains an active research direction for settings in which labeled medical data are limited.

The remainder of this study is organized as follows: Section 2 provides a detailed description of the proposed Self-Supervised Stacked Masked Denoising Autoencoder framework; Section 3 introduces the experimental setup and results analysis; finally, Section 4 concludes the paper and discusses future research directions. The experimental section compares the proposed method with the selected baselines on brain tumor MRI classification and reports the observed performance, ablation behavior, mask-ratio sensitivity, representation characteristics, and noise sensitivity.

## 2. Methods

### 2.1. StackedMDAE Network Architecture

This study develops an integrated Stacked Masked Denoising Autoencoder (StackedMDAE) architecture, designed to learn corruption-aware and discriminative feature representations from brain MRI images through successive self-supervised reconstruction stages. The model consists of a backbone feature extraction network and a classification head. Its architectural role is to transform and reconstruct representations at multiple computational stages; the specific information encoded by each block is treated as an empirical question and is evaluated in the layer-wise representation analysis.

#### 2.1.1. Overall Framework

**Figure 1:**
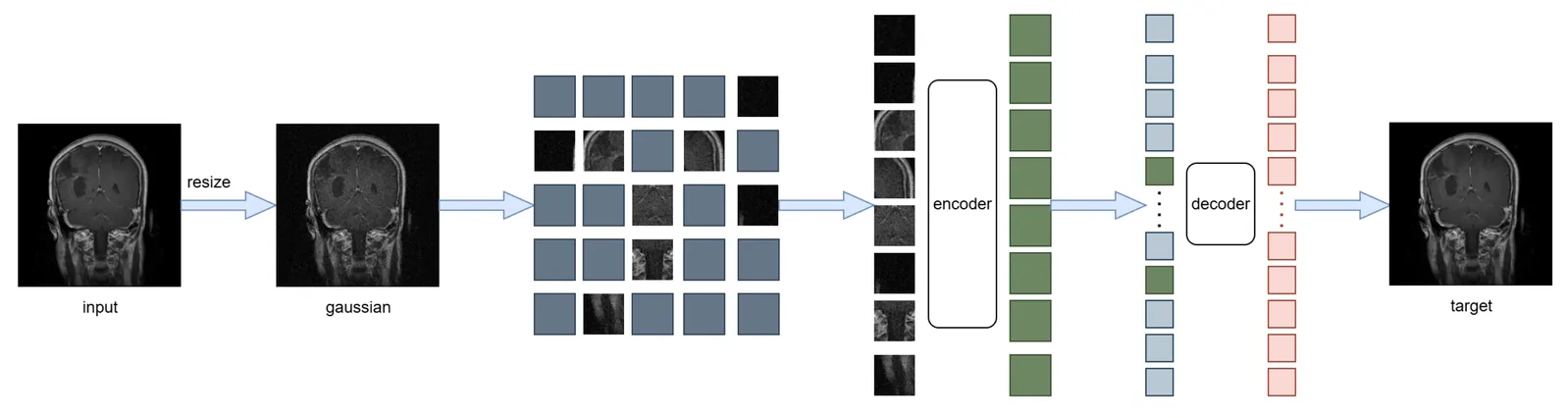
The S^2^MDAE Architecture Proposed in This Paper.

As shown in Figure 1 (network architecture diagram), the StackedMDAE model takes single-channel grayscale MRI images uniformly resized to 224 × 224 as input. The model backbone consists of three sequentially connected MDAE Blocks. Each MDAE block takes the output features from the previous layer as input and produces two parallel outputs: (1) Reconstruction Output, used to reconstruct the masked input regions at the current feature level; (2) Latent Feature Representation, which serves as the input for the next MDAE block. Finally, the feature representation output by the last MDAE block is fed into a lightweight classifier head for tumor classification. This design enables the model to learn general features through reconstruction tasks during the pre-training phase, while allowing these features to serve the final classification objective during the fine-tuning stage.

#### 2.1.2. Architectural Relationship to Stacked Denoising Autoencoders and MAE

The use of multiple encoder layers is not unique to S^2^MDAE. The original stacked denoising autoencoder (SDAE) generally performs greedy layer-wise pre-training: each denoising autoencoder reconstructs a clean input or hidden representation from a corrupted counterpart, after which the trained encoders are stacked and fine-tuned for the downstream task [14]. MAE also uses a deep encoder, but samples a mask once at the image-patch level, encodes the visible tokens, and uses a single lightweight asymmetric decoder to reconstruct the masked pixels; the decoder is discarded for downstream fine-tuning [9].

S^2^MDAE differs in the placement and joint optimization of its reconstruction objectives. Three complete convolutional encoder–decoder blocks are connected sequentially. Each block receives the encoded representation produced by the preceding block, applies an independently generated mask at that feature level, and reconstructs the input representation of the same block. The block-wise reconstruction losses are summed and optimized jointly rather than through a greedy one-block-at-a-time schedule. In addition, Gaussian noise is applied to the image input as a second corruption source, while the final encoded representation is connected to a classification head and the complete encoder stack is fine-tuned end-to-end. Table 1 summarizes these implementation-level distinctions. They define the task-specific architecture evaluated in this work, but do not imply that stacking, denoising, masking, or deep encoders are newly introduced concepts.

#### 2.1.3. MDAE Block Design

**Figure 2:**
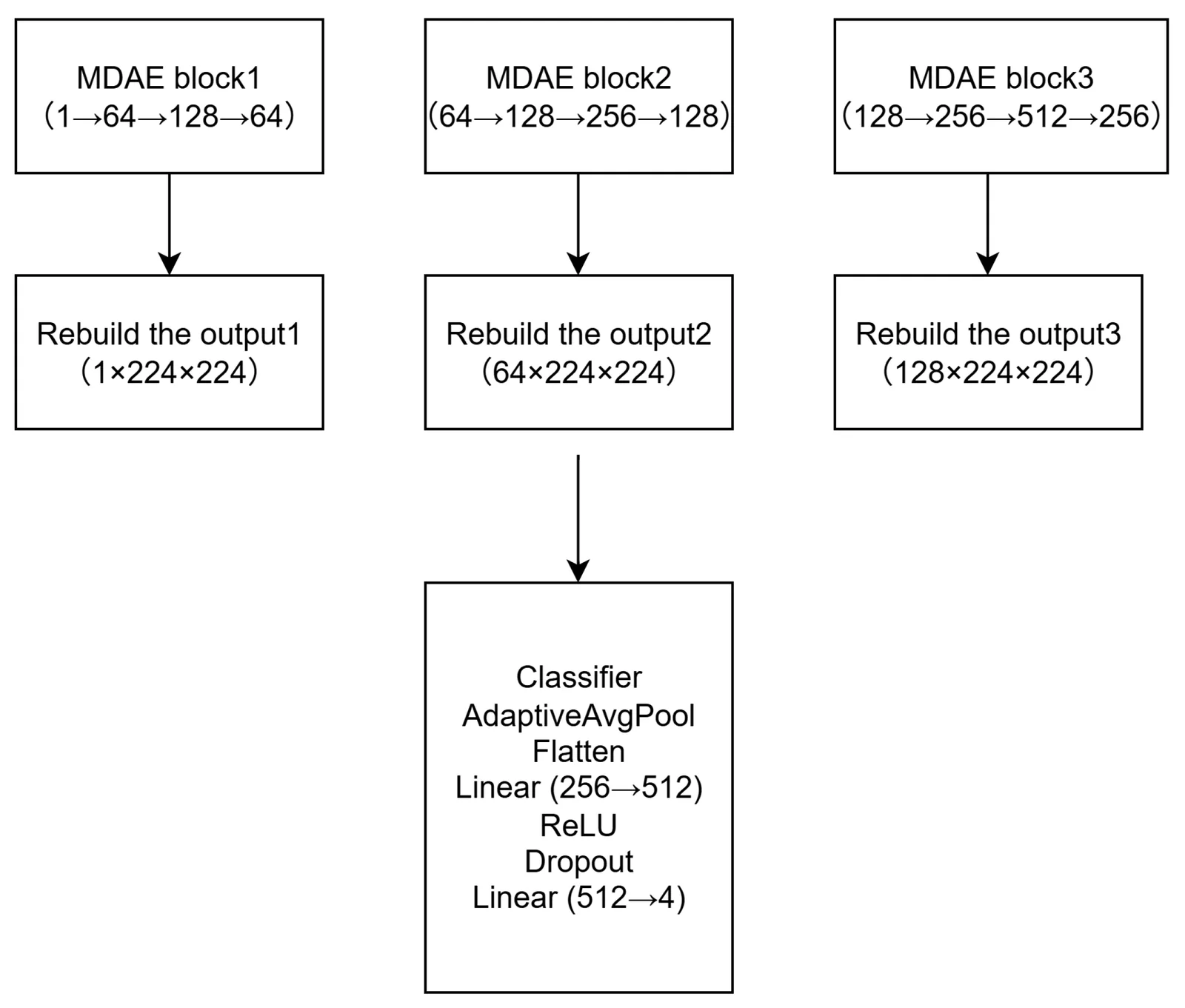
StackedMDAE Network Architecture Diagram.

Each MDAE block functions as a feature learner at its corresponding hierarchical level. As illustrated in Figure 2 (Internal Structure of the MDAE Block), its architecture primarily consists of three components: an Encoder, Mask Tokens, and a Decoder.

Encoder: The encoder $E_{l}$ comprises three convolutional layers (Conv2d), each followed by batch normalization, ReLU activation, and dropout. It maps the input feature map $X_{l}\in R^{C_{\mathrm{in}}\times H\times W}$ to the latent representation $Z_{l}\in R^{D_{l}\times H\times W}$, where $D_{l}$ is the latent dimension of the block. The encoding process is $Z_{l}=E_{l}\left(X_{l}⊙M_{l}\right)$, where ⊙ denotes element-wise multiplication and $M_{l}$ is a binary mask (1 for visible positions and 0 for masked positions). Whereas MAE samples a mask once on image patches before its deep encoder [9], S^2^MDAE samples a separate mask for the representation entering each MDAE block. This “layer-wise masking” implementation is a specific design choice of our integrated framework: reconstruction supervision is applied at each successive block rather than only at the image input. The design supplies multiple learned representations, but it does not by itself establish a predefined detail-to-semantics division among the blocks. This process can be formally understood as constructing a proxy task at each layer, where the encoder learns to map from the partial observation to the complete features. A masking ratio of 0.75 is used as the primary configuration. This high-mask setting was motivated by prior masked autoencoding work, in which substantial masking is used to prevent trivial reconstruction and encourage contextual inference [9]. Because this motivation alone does not establish task-specific optimality, additional experiments at masking ratios of 0.50, 0.60, and 0.90 are reported in Section 3.2.4.

The motivation for applying masks at each block rather than only at the image input is that input-only masking directly constrains only pixel-level recovery. After the first encoder has produced a dense feature map, deeper blocks in an input-only design can process all intermediate activations without having to infer missing feature positions. In the layer-wise configuration, independently sampled masks expose each block to a different partial observation and require its local decoder to reconstruct the representation entering that block. Each stage therefore receives a non-trivial self-supervised signal at its own representation level. This design is intended to encourage contextual inference throughout the stack and reduce dependence on co-adapted activation patterns across successive blocks. These considerations provide an architectural motivation rather than a formal theoretical guarantee; a direct input-only versus layer-wise masking ablation is reported in Section 3.2.3.

Learnable Mask Token: To compensate for the information loss in masked regions, we introduce a learnable vector as the mask token $T_{l}\in R^{D_{l}}$. Unlike methods that use fixed values (such as zero) or projected tokens, our mask token is optimized through gradient descent during training, dynamically learning to represent the “common characteristics” of masked regions at their respective feature hierarchy. The encoder output $Z_{l}$ is combined with the mask $M_{l}$ (downsampled to match the spatial dimensions of $Z_{l}$) to generate the decoder input ${Z_{l}}^{\prime }$: ${Z_{l}}^{\prime }=Z_{l}⊙M_{l}+T_{l}•\left(1-M_{l}\right)$. Here, the • operation denotes broadcasted multiplication: the vector $T_{l}$ is broadcast to the same dimensions as the tensor $1-M_{l}$ before element-wise multiplication is performed. This operation replaces the masked positions in the latent representation with the broadcasted mask token.

Decoder: The decoder $D_{l}\left(\cdot \right)$ features a symmetric structure to the encoder, comprising a sequence of transposed convolutional layers (ConvTranspose2d). Its objective is to reconstruct the layer’s input $X_{l}$ from $Z_{l}^{\prime }$. The final layer of the decoder utilizes a convolutional layer with a Sigmoid activation function to adjust the channel number back to $C_{in}$ and constrain the output values within the [0, 1] range, producing the reconstruction output ${\hat{X}}_{l}$. The reconstruction process is formulated as ${\hat{X}}_{l}=D_{l}\left(Z_{l}^{\prime }\right)$.

Consequently, the MDAE block propagates the latent representation $Z_{l}$ to the subsequent block while outputting the reconstruction result ${\hat{X}}_{l}$ for computing the reconstruction loss.

#### 2.1.4. Classifier Head

After feature extraction through all MDAE blocks, we obtain the final block feature map $Z_{L}$. The classification head takes $Z_{L}$ as input and outputs a 4-dimensional class probability vector (corresponding to the four categories: glioma, meningioma, notumor, pituitary). This head network is composed of a Global Average Pooling layer (AdaptiveAvgPool2d), a Flatten layer, and two Fully Connected layers (Linear), with ReLU activation and Dropout applied in between to prevent overfitting. The computational process is as follows:

(1) ypred=σsoftmax(W2×δdropout(ρReLU(W1×(ϕflatten(ϑGAP(ZL))))))

Here, $W_{1}$ and $W_{2}$ denote the weights of the two fully connected layers.

### 2.2. Two-Phase Training Strategy

To fully leverage the advantages of the StackedMDAE model, a two-phase training strategy was employed: first, self-supervised pre-training, followed by supervised fine-tuning.

#### 2.2.1. Pre-Training Phase

During the pre-training phase, we utilize only unlabeled image data to initialize the model weights through a Masked Image Modeling (MIM) task. We apply Gaussian noise with standard deviation σ=0.1 as an additive corruption regularizer, obtaining the noisy image $X_{noisy}$. This training value is not, by itself, treated as evidence of general robustness; sensitivity to σ∈{0,0.05,0.10,0.20,0.30} is evaluated separately in the experiments. Subsequently, a random binary mask $M_{l}$ is generated for each MDAE block.

The training objective is to minimize the aggregated reconstruction loss from all MDAE blocks. For the *l*-th block, its reconstruction loss $L_{recon}^{l}$ is computed using Mean Squared Error (MSE), considering only the differences in the masked regions $\left(1-M_{l}\right)$:

(2) $$L_{recon}^{l}=\frac{1}{N}\sum_{i}\left|\right|\left(\hat{X_{l}^{i}}-X_{l}^{i}\right)⊙\left(1-M_{l}^{i}\right){\left|\right|}_{2}^{2}$$

where *N* is the number of samples. For the first block, $X_{l}$ is the original clean image; for each subsequent block, $X_{l}$ is the feature output $Z_{l-1}$ from the preceding block. The decoder output channels are defined to match the corresponding block input channels, so the reconstruction target and output are dimensionally aligned by construction. The total pre-training loss is $L_{\mathrm{pretrain}}=\sum_{l=1}^{L}L_{\mathrm{recon}}^{l}$. We use the Adam optimizer and a ReduceLROnPlateau scheduler based on reconstruction loss measured on the released evaluation partition.

#### 2.2.2. Fine-Tuning Phase

Upon completion of pre-training, we load the pre-trained weights and attach the classification head to the top layer. Subsequently, the entire network undergoes end-to-end supervised fine-tuning using labeled data. The training objective in this phase is to minimize the cross-entropy loss for the classification task:

(3) $$L_{cls}=-\frac{1}{N}\sum_{i=1}^{N}\sum_{c=1}^{C}y_{i,c}\times \log\left({\hat{y}}_{i,c}\right)$$

where $y_{i,c}$ is the ground-truth class label (one-hot encoded) of sample *i*, ${\hat{y}}_{i,c}$ is the predicted class probability, and C=4. During this phase, we employ a smaller learning rate to stabilize training and prevent the disruption of the well-learned features acquired during pre-training.

## 3. Experiment

### 3.1. Experimental Setup

#### 3.1.1. Dataset and Evaluation Metrics

This study uses the publicly available Brain Tumor MRI Dataset, which contains four categories: glioma, meningioma, pituitary tumor, and no tumor. The released dataset provides two official partitions rather than three independent partitions: a Training partition containing 5712 images and a Testing partition containing 1311 images. In our local implementation, the folder corresponding to the official Testing partition was named “val”; it is not a separate third validation cohort. The Training partition was used for parameter optimization, whereas the official Testing partition was used for epoch-wise monitoring, checkpoint selection, and reporting the primary-dataset metrics. Accordingly, this partition is described as the released evaluation partition rather than as a completely untouched independent test set. This protocol is stated explicitly as a limitation.

The complete class-wise sample distribution is summarized in Table 2. The imbalance is moderate rather than extreme: the largest-to-smallest class ratios are 1.21 in the training partition and 1.35 in the test partition.

#### 3.1.2. External Dataset and Cross-Dataset Screening

To evaluate cross-dataset generalization, we additionally used the public Brain Tumor MRI Dataset (Glioma, Meningioma, Pituitary, No Tumor), Version 4, released through Mendeley Data [18]. It contains 12,064 pre-processed T1-weighted contrast-enhanced brain MRI images organized into training and testing folders with the same four diagnostic categories as the primary dataset. Only the external Test split was used in this study; no image from the external dataset was used for model training, model selection, or fine-tuning.

Because publicly assembled brain MRI datasets may share source images, we screened the complete external Test split against all 7023 images in the primary dataset before evaluation. Exact duplicates were checked using MD5 content hashes, and visually near-duplicate images were checked using a 64-bit perceptual hash with a Hamming-distance threshold of 4. The initial external Test split contained 2414 images. No exact MD5 duplicates were found, while 87 perceptually near-duplicate images were removed. The resulting external evaluation set contained 2327 images: 727 glioma, 514 meningioma, 469 no-tumor, and 617 pituitary images. This duplicate-screened set was used for all external results.

The checkpoints trained exclusively on the primary dataset were evaluated directly on the external set without retraining or adaptation. We evaluated the full S^2^MDAE model, the no-pretraining ablation, and the CNN baseline. In addition to Accuracy, Balanced Accuracy and macro-averaged metrics were reported to account for the external class distribution. Ninety-five percent confidence intervals for Accuracy and Macro-F1 were estimated using 1000 bootstrap resamples.

#### 3.1.3. Implementation Details

The model was implemented in PyTorch (Python 3.12.9) and trained on an NVIDIA RTX 4090 GPU. Input images were converted to grayscale, resized to 224 × 224, and augmented via random horizontal flipping and rotation (within 10°). During pre-training, we used a batch size of 16, the Adam optimizer (lr = 1 × 10^−5^, weight decay = 1 × 10^−5^), and trained for 30 epochs. For fine-tuning, we maintained the batch size, reduced the learning rate to 5 × 10^−6^, and trained for 50 epochs. The 5712-image Training partition was used for model optimization. Because the released dataset does not contain a separate validation partition, the 1311-image official Testing partition was used for model monitoring and checkpoint selection as well as final primary-dataset reporting. This limitation is considered when interpreting the reported primary-dataset performance.

The classifier processes the 256-channel feature map produced by the third MDAE block. Its structure consists sequentially of a global average pooling layer, a flatten layer, two fully connected layers, and an output layer, optimized with cross-entropy loss to map the learned representation to class predictions. No class weighting or over-sampling is applied. To reduce the influence of the moderate class-count differences on interpretation, we report macro-averaged Precision, Recall, and F1-score in addition to overall Accuracy.

### 3.2. Results

#### 3.2.1. Main Results

Table 3 consolidates the performance under the three evaluated data settings.

The original dataset provides the highest overall scores, whereas the proposed setting achieves 87.207% Accuracy and maintains balanced macro-averaged Precision, Recall, and F1-score on the noisy MRI data. The unprocessed noisy setting yields the lowest values among the three conditions, indicating that the adopted training strategy improves classification performance under the evaluated noisy-data condition.

#### 3.2.2. Ablation Studies

To further clarify the contribution of each component, we systematically ablated three key designs: pre-training (no pretrain), the masking mechanism (no mask), and noise injection (no noise), comparing their performance on the same test set. Additionally, we compared our method with a standard CNN baseline and a ResNet50 model trained without our proposed pre-training strategy to comprehensively evaluate the effectiveness of our approach.

Table 4 consolidates the ablation and baseline comparisons. The complete model (S^2^MDAE) achieves the highest Accuracy and macro-averaged F1-score among all evaluated variants. Specifically: No Pretraining: When the model is trained only with supervised classification, accuracy decreases from 87.207% to 80.778%. This result indicates that the self-supervised pretraining loss $L_{pretrain}=\sum_{l=1}^{L}L_{recon}^{l}$ contributes to the reported performance in this experimental setting. Without reconstruction-based pretraining, the model does not benefit from initialization learned from masked and noisy inputs. Lower recall is observed for the meningioma and glioma categories.

Comparative Analysis of No-Mask and No-Noise Ablations: Removing the masking mechanism is associated with an accuracy decrease to 77.193%, indicating that the masked reconstruction objective $\frac{1}{N}\sum_{i}\left|\right|\left(\hat{X_{l}^{i}}-X_{l}^{i}\right)⊙\left(1-M_{l}^{i}\right){\left|\right|}_{2}^{2}$ contributes to context-dependent representation learning in the evaluated configuration. Removing noise injection reduces accuracy to 84.134%, indicating a contribution from the corruption component to the overall classification result. This ablation alone does not establish robustness across noise intensities; that question is examined separately through the Gaussian-noise sensitivity analysis. Together, the ablations quantify the effects of masking and noise without assigning exclusive low-level or high-level functions to individual blocks.

Comparison with Baseline Models: The standard CNN obtains an accuracy of 64.683%, while ResNet50 without pretraining obtains 84.897%, compared with 87.207% for S^2^MDAE. These results show higher measured accuracy for the proposed configuration than for the two evaluated supervised baselines.

In summary, the ablation studies comprehensively validate the necessity of the three key components—pretraining, the masking mechanism, and noise injection—in the S^2^MDAE model, as well as their positive impact on final performance. These components work jointly to enable the model to learn discriminative and corruption-aware representations, thereby achieving the best performance among the evaluated variants in the brain tumor MRI classification task.

#### 3.2.3. Layer-Wise Mask Placement Ablation

To isolate the effect of mask placement, we conducted a controlled ablation comparing the proposed layer-wise independent masking strategy with an input-layer-only strategy. Both variants used the same dataset split, three-block architecture, 75% mask ratio, Gaussian-noise level, initialization seed, pre-training and fine-tuning epochs, learning rates, and optimization settings. In the layer-wise variant, an independent mask was sampled for each MDAE block and masked reconstruction loss was applied at all three representation levels. In the input-only variant, only the first block received a random mask; the second and third blocks received all-one masks and did not contribute masked reconstruction losses. Thus, the comparison changes the placement of masking and the associated block-wise masked reconstruction supervision while retaining the remaining training protocol. The results of this controlled comparison are summarized in Table 5.

Layer-wise independent masking improves Accuracy from 65.828% to 71.777%, an increase of 5.950 percentage points, and improves Macro-F1 from 64.293% to 69.300%, an increase of 5.007 percentage points. Balanced Accuracy and Weighted-F1 also increase by 4.765 and 5.249 percentage points, respectively. The class-wise reports show that the largest improvements occur for the no-tumor and pituitary categories, while glioma F1 remains nearly unchanged. These results support the empirical value of introducing non-trivial masked reconstruction objectives at successive representation levels rather than limiting masking to the image input. However, because this is a controlled single-seed ablation, it demonstrates an empirical performance advantage in the evaluated setting rather than proving a general theoretical property.

#### 3.2.4. Mask-Ratio Sensitivity Analysis

The primary model uses a 75% masking ratio. To examine whether the classification result is sensitive to this design choice, we additionally trained models with masking ratios of 50%, 60%, and 90%. These supplementary runs use the same dataset split, architecture, pre-training and fine-tuning epoch counts, learning rates, Gaussian-noise level, and optimization objective as one another. The 75% result remains the primary result reported in Table 3, whereas the results for the three alternative mask ratios are summarized in Table 6. These additional runs are presented as a sensitivity check rather than as a replacement for the main experiment.

Among the three alternative settings, the 60% mask ratio gives the highest Accuracy (71.014%) and Macro-F1 (69.420%), followed by 90% and 50%. The results show that classification performance is sensitive to the masking ratio rather than invariant to this hyperparameter. A plausible interpretation is that 50% masking may provide a less demanding reconstruction task, whereas 90% masking may remove excessive information from small and low-contrast MRI structures. These explanations are treated as hypotheses rather than directly measured mechanisms. Together with the primary 75% configuration, the supplementary results support the use of a high but non-extreme masking ratio in the evaluated task; they do not establish 75% as a universally optimal value.

#### 3.2.5. Gaussian-Noise Sensitivity Analysis

To evaluate robustness beyond the single training corruption level of σ=0.1, we conducted a controlled sensitivity analysis at σ∈{0,0.05,0.10,0.20,0.30}. Zero-mean Gaussian noise was added dynamically to the clean official Testing images. The full S^2^MDAE and the no-noise ablation model were evaluated using identical corrupted inputs. Each non-zero condition was repeated with five deterministic noise realizations, and the mean and standard deviation were calculated. Because robustness concerns degradation relative to an uncorrupted reference, we report performance retention, defined as $R_{m}\left(\sigma \right)=m\left(\sigma \right)/m\left(0\right)\times 100\%$, where *m* denotes Accuracy or Macro-F1. This normalization characterizes each model’s relative degradation under the controlled corruption protocol and does not replace the absolute classification accuracies reported in the main ablation experiment. The resulting Accuracy and Macro-F1 retention values are summarized in Table 7.

**Figure 3:**
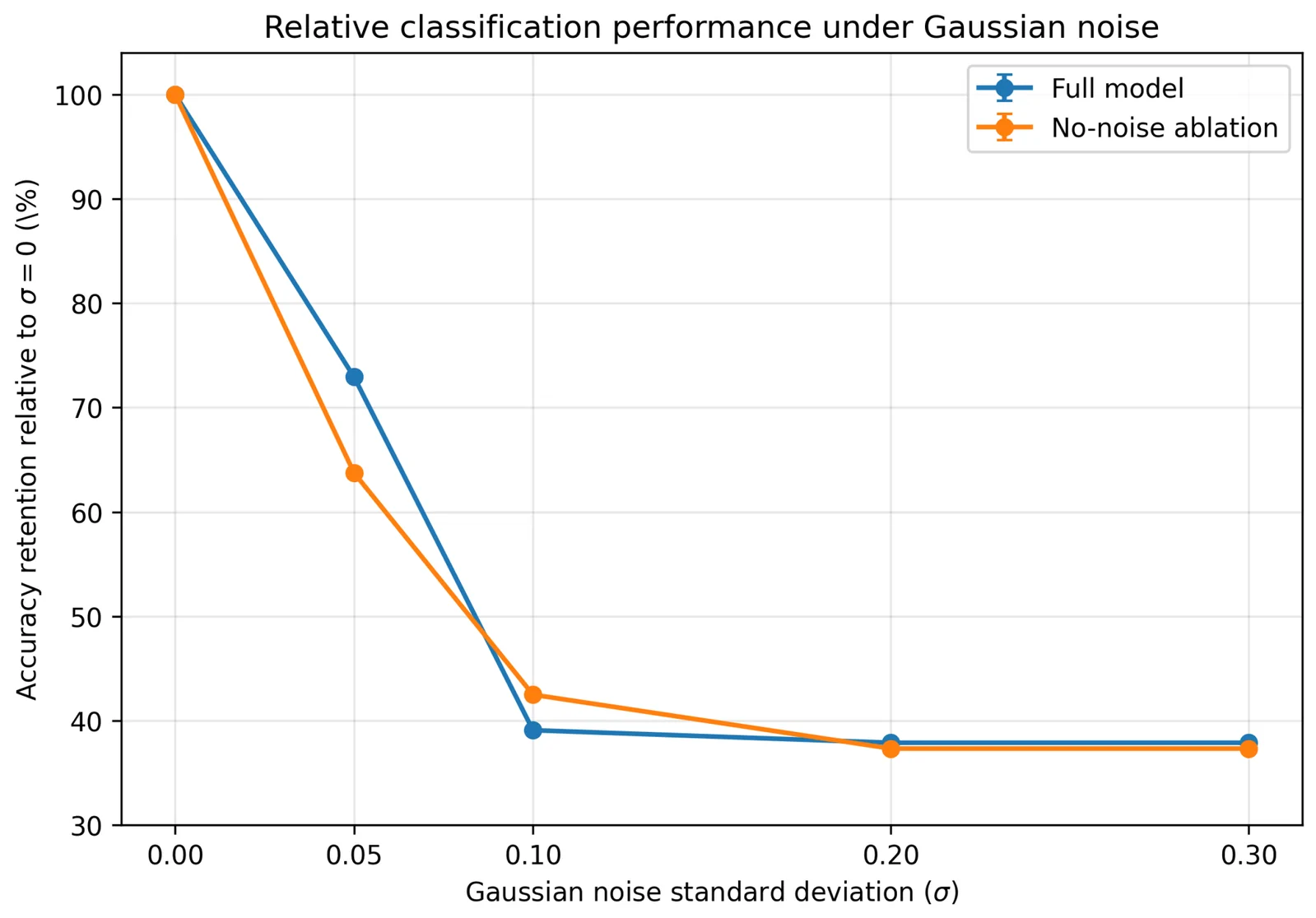
Accuracy retention under increasing Gaussian-noise intensity. Each curve is normalized to the corresponding model’s own σ=0 Accuracy. Error bars denote the standard deviation over five deterministic noise realizations for non-zero σ.

At σ=0.05, the full model retains 72.93% of its clean-condition Accuracy, compared with 63.74% for the no-noise ablation, a retention advantage of 9.19 percentage points. Macro-F1 retention shows the same mild-noise advantage (60.78% versus 43.73%). This advantage is not monotonic: at σ=0.10, the no-noise ablation retains more Accuracy and Macro-F1 than the full model, and at σ≥0.20 both models approach similarly low retention levels. The results therefore demonstrate limited tolerance to mild Gaussian perturbation rather than broad robustness across corruption intensities. The corresponding Accuracy-retention curves across the evaluated Gaussian-noise intensities are shown in Figure 3.

This controlled experiment evaluates downstream classification sensitivity to synthetically added Gaussian noise; it is not an evaluation of clinical denoising quality on genuinely low-SNR acquisitions. The available public datasets contain already reconstructed images and do not provide raw k-space data, acquisition parameters, repeated acquisitions, noise maps, SNR annotations, or paired low- and high-SNR references. Consequently, a real low-SNR subgroup cannot be identified reliably, and image-restoration metrics such as PSNR or SSIM cannot be computed against a valid clean reference. We therefore do not infer “real low-SNR” status from visual appearance or post hoc image-intensity statistics. Validation on prospectively or retrospectively curated low-SNR MRI data with acquisition metadata and, where possible, matched higher-SNR references is left for future work.

#### 3.2.6. Comparison Scope and Fairness

To reduce the influence of backbone differences, we conducted a controlled comparison in which S^2^MDAE, SimCLR [11], BYOL [12], and MoCo v2 [13] used the same three-stage stacked convolutional encoder, classification head, dataset split, training budget, and evaluation procedure. Under this matched setting, S^2^MDAE achieved 87.207% Accuracy and 86.142% Macro-F1, compared with 64.989% and 62.948% for SimCLR, 65.980% and 61.676% for BYOL, and 69.336% and 65.867% for MoCo v2, respectively. These results show that the proposed pre-training objective achieves the strongest overall Accuracy and Macro-F1 under the controlled encoder setting. For broader comparison, we also report the results obtained using the native Transformer architectures of MAGE [7] and I-JEPA [8], which achieved 76.049% Accuracy and 73.919% Macro-F1, and 66.895% Accuracy and 64.964% Macro-F1, respectively. Because their Transformer-based designs are integral to the original methods, these values are presented as complementary cross-architecture references rather than as part of the matched-backbone comparison.

#### 3.2.7. Visualization and Analysis

**Figure 4:**
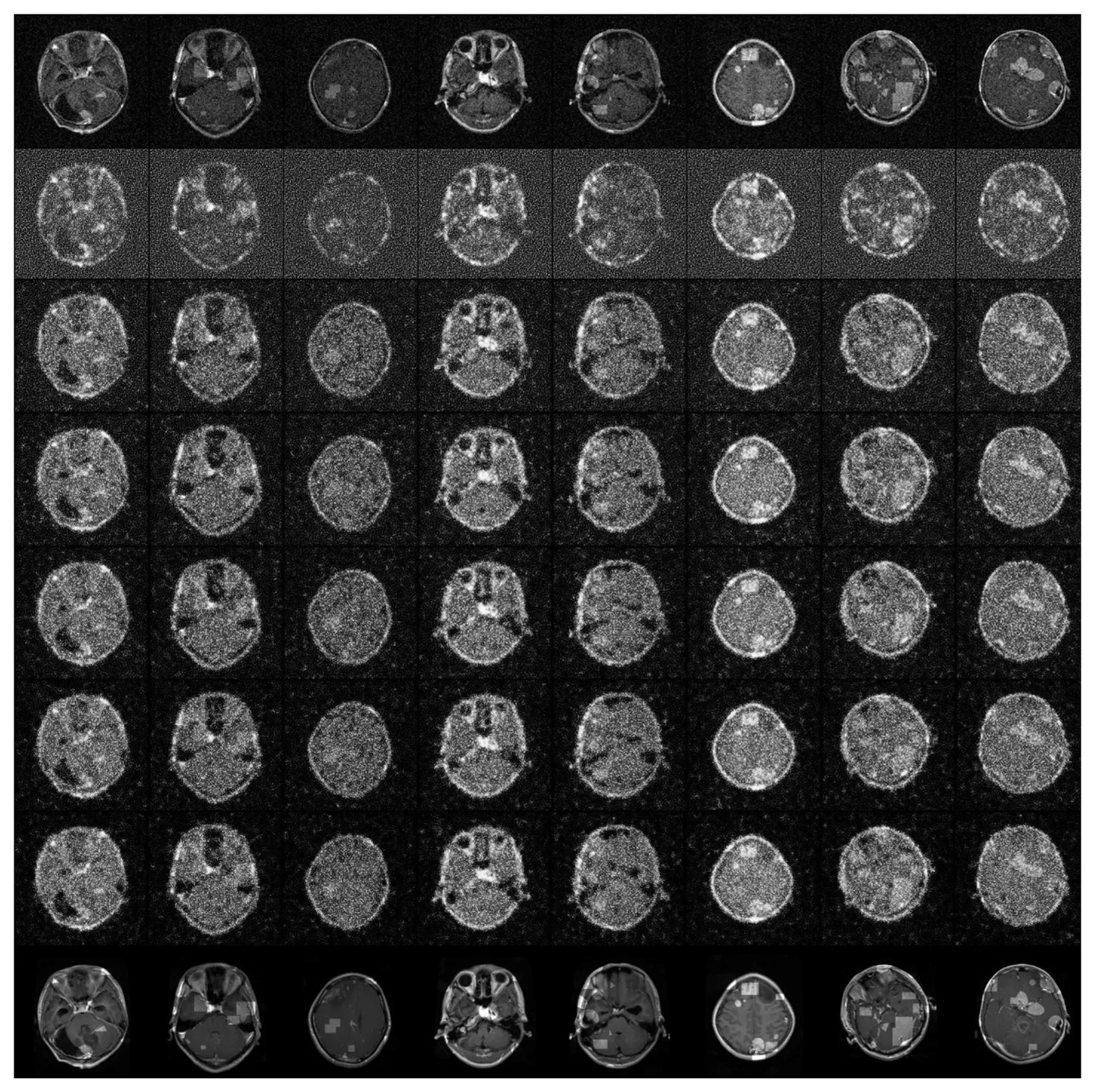
Process images of different rounds.

Figure 4 visualizes reconstruction outputs at different training epochs. Early reconstructions are blurred and mainly preserve coarse anatomical structure, whereas later reconstructions contain sharper boundaries and more local detail. This figure demonstrates progressive optimization of the reconstruction objective over epochs. Importantly, it compares training epochs rather than intermediate MDAE blocks and therefore cannot, by itself, establish that lower blocks specialize in details or that upper blocks specialize in semantics.

**Figure 5:**
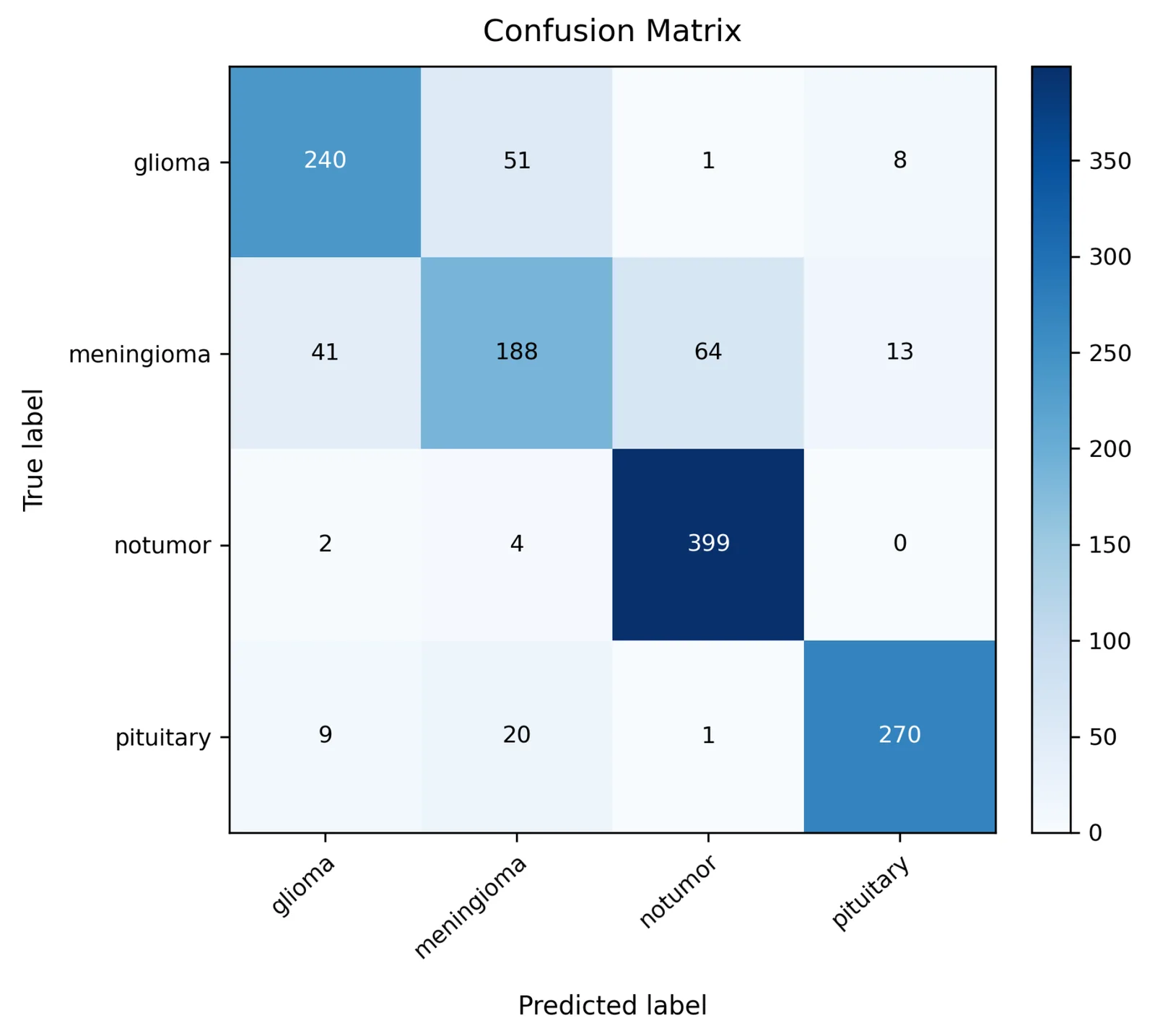
Confusion matrix for the four-class brain MRI classification task. Rows indicate the true classes and columns indicate the predicted classes.

Figure 5 provides task-level evidence from the downstream classifier. The model achieves high accuracy across the four classes, while the main confusion occurs between glioma and meningioma. This result demonstrates discriminative utility of the final representation, but it does not resolve the functional content of each intermediate block.

In summary, the reconstruction and classification visualizations demonstrate optimization of the pretext task and discriminative utility of the final representation. Direct block-wise evidence is provided separately through the layer-wise representation analysis below.

#### 3.2.8. Layer-Wise Representation Analysis

To directly examine the representations produced by the three MDAE blocks, we extracted the output of each block from both the self-supervised pre-trained checkpoint and the supervised fine-tuned checkpoint. Global average pooling converted the block outputs into 64-, 128-, and 256-dimensional vectors, respectively. For each block, a multinomial logistic-regression linear probe was fitted on the pooled training representations and evaluated on the official Testing representations. We additionally calculated the Silhouette Score on the official Testing features and visualized the representations using t-SNE. The analysis used clean images from the same Training and official Testing partitions as the main experiment.

As shown in Table 8, the pre-trained representations do not exhibit a monotonic improvement with depth: Block 1 obtains the highest pre-trained linear-probe accuracy (78.719%), while Blocks 2 and 3 achieve lower scores and all three pre-trained Silhouette Scores are negative. After supervised fine-tuning, however, class separability increases across the successive blocks. Linear-probe accuracy rises from 77.727% at Block 1 to 80.931% at Block 2 and 81.846% at Block 3; the corresponding Silhouette Score increases from 0.0147 to 0.0217 and 0.0631. Thus, the evidence supports stage-dependent representational differentiation and a fine-tuning-induced improvement in deeper-block class separability, rather than a universal or predetermined detail-to-semantics hierarchy.

**Figure 6:**
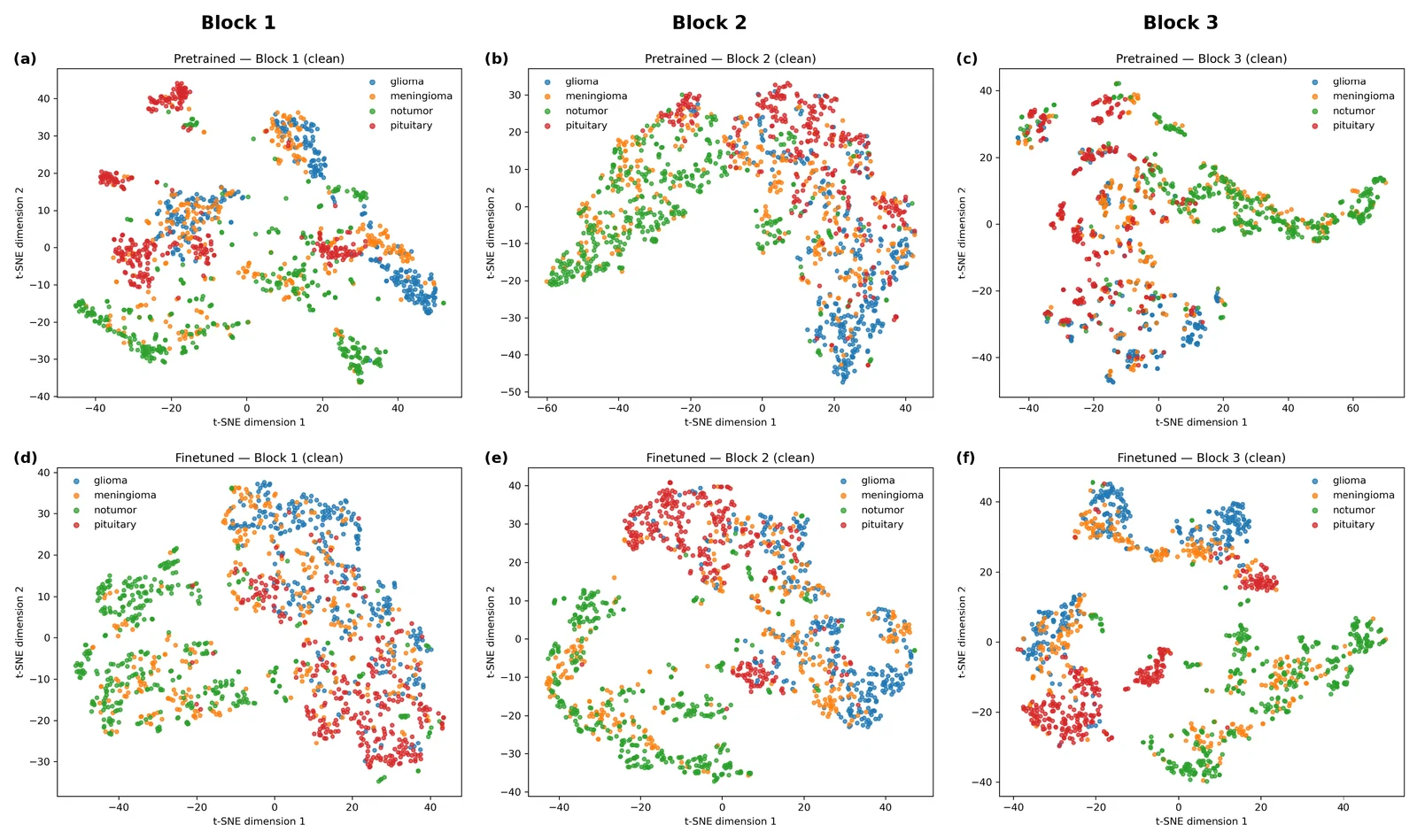
t-SNE visualizations of pooled block representations. (**a**–**c**) Pre-trained representations from Blocks 1–3; (**d**–**f**) fine-tuned representations from Blocks 1–3. Fine-tuning produces clearer class organization in the intermediate and final blocks, although overlap remains, particularly between glioma and meningioma.

The t-SNE visualization in Figure 6 is consistent with the quantitative analysis. The pre-trained feature spaces contain substantial class overlap, whereas the fine-tuned Block 2 and Block 3 spaces show clearer class organization. Because t-SNE is a nonlinear projection, this figure is used only as qualitative support; the linear-probe and Silhouette results provide the primary quantitative evidence.

**Figure 7:**
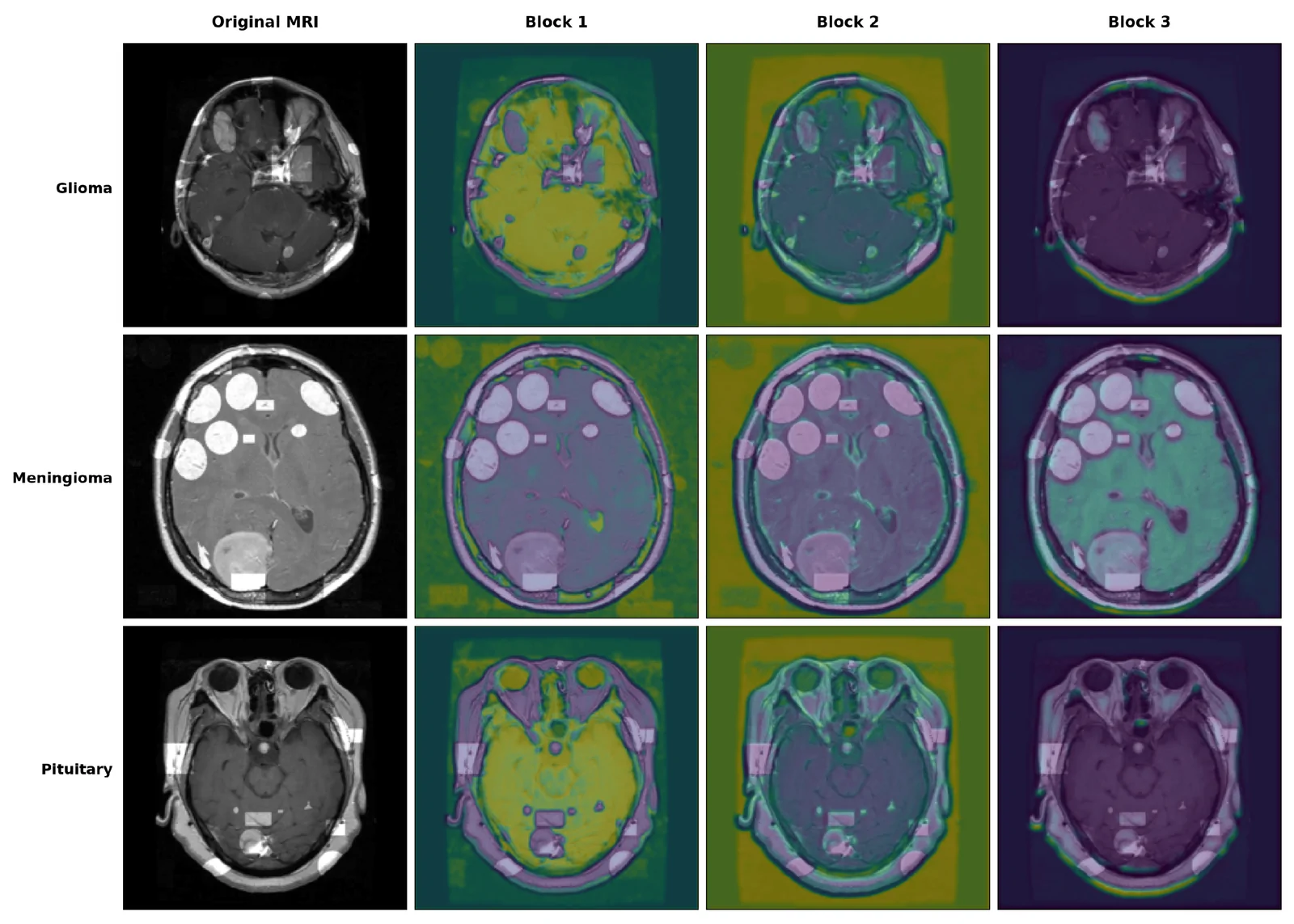
Representative layer-wise mean absolute channel-response maps from the fine-tuned model for glioma, meningioma, and pituitary MRI images. The maps illustrate spatial redistribution of activation across Blocks 1–3. They are descriptive activation summaries and should not be interpreted as lesion-localization maps or as direct proof of semantic specialization.

Figure 7 provides direct block-wise visualization. The activation distributions change substantially across the three blocks, confirming that the successive modules transform the spatial response patterns. Nevertheless, mean channel responses do not identify the semantic content encoded by a block and are not equivalent to class-discriminative attention or Grad-CAM. Accordingly, we restrict our conclusion to observable representational differentiation and class separability.

#### 3.2.9. Cross-Dataset External Generalization

Table 9 reports the strict external evaluation results after cross-dataset duplicate screening. The full S^2^MDAE achieves 67.254% Accuracy (95% CI: 65.320–69.102%), 67.149% Balanced Accuracy, and 66.009% Macro-F1 (95% CI: 64.076–67.784%). Without any external retraining or fine-tuning, it exceeds the no-pretraining ablation by 2.922 percentage points in Accuracy and 3.658 percentage points in Macro-F1. It also exceeds the CNN baseline by 23.206 percentage points in Accuracy and 23.953 percentage points in Macro-F1. These comparisons indicate that the self-supervised pretraining stage contributes to transfer across the evaluated dataset shift.

The external dataset was evaluated without retraining, fine-tuning, or model selection on external images. Under this strict protocol, S^2^MDAE retains a clear advantage over the no-pretraining and CNN baselines, demonstrating practical transferability of the learned representation across datasets. The class-wise F1-scores are 83.675% for pituitary tumors, 68.343% for glioma, 66.139% for no tumor, and 45.877% for meningioma. The strong pituitary performance and stable glioma and no-tumor recognition show that useful discriminative information is preserved under dataset shift, while the comparatively lower meningioma score provides a focused target for further refinement.

#### 3.2.10. Predictive Uncertainty and Calibration Considerations

The confidence intervals reported for the external Accuracy and Macro-F1 quantify sampling uncertainty in aggregate performance metrics. Similarly, the variability across repeated synthetic-noise realizations characterizes sensitivity to a specified corruption process. Neither analysis, however, estimates uncertainty for an individual diagnostic prediction or determines whether the softmax probabilities are calibrated. The present S^2^MDAE is a deterministic classifier and does not explicitly separate aleatoric uncertainty arising from ambiguous or noisy images from epistemic uncertainty arising from limited training data or model uncertainty.

Recent magnetic-resonance studies illustrate several complementary approaches. Küstner et al. combined deep ensembles with an uncertainty-aware objective to estimate epistemic and aleatoric components in deep-learning-based MRI reconstruction and evaluated their behavior under domain shifts [19]. Ekanayake et al. proposed PixCUE, which produces a reconstruction and a pixel-level uncertainty map in a single forward pass and demonstrated correlations between estimated uncertainty and reconstruction error [20]. In multidimensional NMR spectroscopy, Zhan et al. compared Deep Ensemble, Monte Carlo Dropout, and Evidential Deep Learning frameworks and emphasized that reliable prediction should be accompanied by uncertainty estimates rather than a single deterministic output [21]. These studies concern reconstruction or spectroscopy rather than four-class MRI diagnosis, but they establish relevant principles for reliability assessment.

For the present classification setting, a complete uncertainty evaluation would require repeated stochastic or ensemble predictions and an assessment of both discrimination and calibration. Appropriate extensions include Deep Ensembles or Monte Carlo Dropout for epistemic uncertainty, an evidential or heteroscedastic classification head for data-related uncertainty, and evaluation with negative log-likelihood, Brier score, expected calibration error, reliability diagrams, and risk–coverage curves. Comparing these measures between the primary and external datasets would also help determine whether uncertainty increases under domain shift and whether high-uncertainty cases could be referred for expert review. Because these components were not part of the original deterministic architecture and no calibrated uncertainty model was trained, we do not interpret the current maximum softmax probability as a validated confidence estimate.

### 3.3. Analysis

This chapter presents an in-depth analysis of the experimental results of the proposed S^2^MDAE model and conducts a comparative discussion with other methods to elucidate the underlying reasons for its performance advantages.

#### 3.3.1. Performance Advantage Analysis

The performance of S^2^MDAE stems from its combined architecture and pre-training design, enabling feature learning from limited annotated data.

First, dual-corruption pre-training contributes to the classification result. The primary configuration uses 75% random masking to impose a high-context reconstruction task, while additive Gaussian noise provides corruption-based regularization. The added mask-ratio sensitivity analysis shows that performance changes across the 50%, 60%, and 90% alternatives, with 60% performing best among those three settings. This supports treating the mask ratio as a task-dependent hyperparameter rather than an arbitrary or universally fixed constant. The multi-intensity noise experiment further shows improved relative performance retention under mild corruption (σ=0.05), but the advantage is not maintained consistently at higher noise levels. We therefore interpret the noise component as providing limited mild-noise tolerance rather than general robustness to Gaussian corruption.

Second, the controlled mask-placement ablation directly tests why masks are introduced at successive blocks. Under the same seed and training protocol, layer-wise independent masks achieve 71.777% Accuracy and 69.300% Macro-F1, compared with 65.828% and 64.293% for input-layer-only masking. The 5.950-point Accuracy gain and 5.007-point Macro-F1 gain indicate that distributing non-trivial masked reconstruction objectives across the stack is more effective than constraining only the image input in this experiment. The result provides empirical support for the design motivation, while not constituting a formal theoretical proof.

Third, the stacked encoder-decoder architecture provides multiple reconstruction-supervised representation stages. The new layer-wise analysis shows that the pre-trained blocks do not follow a monotonic depth-wise pattern, whereas supervised fine-tuning increases linear class separability from Block 1 to Block 3. We therefore interpret the blocks as stage-dependent transformations and do not assign exclusive detail or semantic functions to specific depths.

The external evaluation provides complementary evidence beyond the primary dataset. The full model retains a measurable advantage over both the no-pretraining ablation and the CNN baseline when all checkpoints are transferred without adaptation to the duplicate-screened external dataset. At the same time, the decrease from the primary-dataset Accuracy to 67.254% externally indicates sensitivity to dataset and acquisition shifts. We therefore interpret the experiment as evidence of partial cross-dataset transfer rather than complete or clinical-level generalization.

#### 3.3.2. Failure Cases, Difficult MRI Images, and Class Distribution

The confusion matrix in Figure 5 shows that the errors are not uniformly distributed across classes. Meningioma is the most difficult category in this evaluation: 188 of 306 images are correctly classified (61.44% recall), while 41 are predicted as glioma, 64 as no tumor, and 13 as pituitary tumor. Glioma achieves 80.00% recall (240 of 300), with 51 cases misclassified as meningioma. In contrast, the no-tumor and pituitary classes achieve recalls of 98.52% (399 of 405) and 90.00% (270 of 300), respectively. The principal failure pattern is therefore the confusion involving glioma and meningioma, together with a subset of meningioma images predicted as no tumor.

A plausible explanation is that individual two-dimensional MRI slices can contain limited lesion coverage, low tumor-to-background contrast, diffuse or heterogeneous appearance, and overlapping intensity or morphological characteristics across tumor types. Noise and acquisition variation can further obscure weak boundaries. These factors are presented as likely sources of difficulty rather than directly annotated causes, because the dataset does not provide lesion-size, contrast, slice-position, or artifact-severity labels for a controlled error-stratification analysis. Consequently, the confusion matrix identifies where the model fails, but it cannot by itself determine the causal imaging characteristic responsible for each error.

The dataset also contains moderate class-count differences. The no-tumor category accounts for 27.92% of the training split and 30.89% of the test split, whereas the other test classes each account for approximately 22.88–23.34%. Although this imbalance is not severe, the larger no-tumor group may influence the learned decision boundaries and the overall Accuracy. We therefore report macro-averaged metrics, which weight all four classes equally, alongside Accuracy. Nevertheless, because no class reweighting or resampling was applied, the present experiment cannot fully separate intrinsic visual difficulty from the effect of class frequency. Future work will evaluate class-balanced or focal losses, controlled resampling, hard-example mining, and performance stratification by lesion size, contrast, slice position, and artifact severity.

#### 3.3.3. Essential Differences from Traditional Methods

As detailed in Section 2, the essential architectural difference from SDAE and MAE is not the presence of multiple encoder layers. It is the combination of independently masked stacked encoder–decoder blocks, joint image- and feature-level reconstruction supervision, dual masking/noise corruption, and end-to-end transfer of the complete encoder stack to classification. SDAE and MAE remain the established methodological foundations on which this task-specific design is built [9,14].

The proposed S^2^MDAE framework also differs from traditional two-stage approaches of “denoising followed by classification” in its optimization objective and workflow. Conventional methods [22,23,24,25,26] (e.g., filter-based or sparse representation denoising algorithms) focus solely on pixel-level restoration, and their optimization objectives (such as PSNR and SSIM) are not directly linked to the final classification performance. A visually “cleaner” image does not necessarily yield more useful classification features and may remove subtle textures that are potentially relevant to diagnosis.

**Figure 8:**
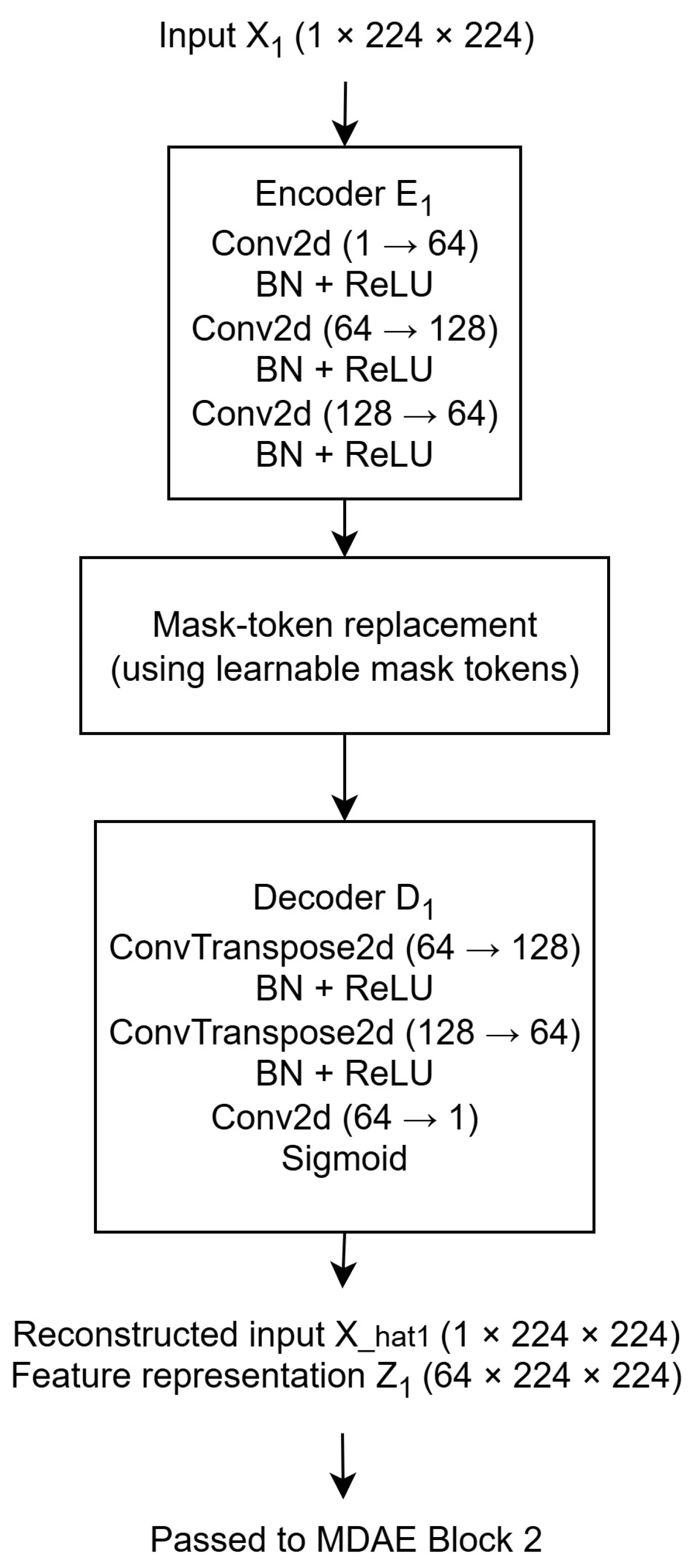
Internal Structure of the MDAE Block (Using MDAE Block 1 as an Example).

The practical distinction of our framework lies in unifying denoising-oriented corruption handling and feature learning within an end-to-end self-supervised pipeline. Rather than performing explicit image restoration as a separate preprocessing stage, the model learns discriminative features through masked reconstruction of noise-corrupted inputs. As visualized in Figure 8, the model learns to “understand” image content beyond merely “repairing” it. This ensures the learned features are optimized for high-level semantic tasks like classification from the outset. The revised controlled comparison no longer uses MAGE or I-JEPA numerical results to support a fairness claim because their native Transformer architectures are integral to their formulations. Under the architecture-matched comparison with SimCLR, BYOL, and MoCo v2, the proposed objective achieves the highest Accuracy and Macro-F1, while the separate corruption analysis indicates only limited tolerance to mild Gaussian noise.

#### 3.3.4. Limitations and Future Work

Despite its promising performance, S^2^MDAE has limitations. First, the primary dataset provides no separate validation partition. The official Testing partition was used for model monitoring and checkpoint selection as well as final primary-dataset reporting, which may introduce optimistic selection bias. Future studies should reserve an untouched test set after creating a development/validation split from the Training partition. Second, although a second public dataset has been added for strict external evaluation, the external Accuracy of 67.254% is substantially lower than the primary-dataset result. The experiment therefore demonstrates partial cross-dataset transfer rather than broad generalization across institutions, scanners, populations, or acquisition protocols. Validation on additional patient-level, multi-center, and prospectively collected cohorts remains necessary. The stacked architecture also increases parameters and training time, which may hinder deployment in resource-constrained settings. In addition, the mask-ratio analysis is a supplementary single-run sensitivity check rather than a multi-seed hyperparameter optimization. Although it demonstrates that performance changes across the evaluated alternatives, broader conclusions about the optimal ratio require repeated training with additional seeds and datasets. Fourth, the Gaussian-noise sensitivity analysis demonstrates only limited tolerance to mild additive corruption, and performance degrades markedly at stronger noise levels. This experiment measures classification sensitivity under synthetic corruption rather than restoration quality on genuinely low-SNR MRI acquisitions. The available datasets lack raw k-space data, acquisition parameters, SNR labels, and paired low-/high-SNR references, so real low-SNR validation and reference-based denoising metrics could not be performed reliably. Future work should evaluate prospectively or retrospectively curated low-SNR MRI data with appropriate acquisition metadata and should additionally consider Rician noise, motion artifacts, bias-field inhomogeneity, blur, and scanner-dependent shifts. Fifth, the added linear probes, clustering metrics, t-SNE plots, and mean activation responses characterize class separability and spatial response changes, but they do not provide a causal or exhaustive account of the information encoded by each block. Future studies should incorporate representational-similarity analysis, controlled concept probing, and class-discriminative localization methods. Sixth, the current failure analysis is based on class-level confusion rather than expert-annotated difficult-case attributes. The moderate class imbalance and the absence of lesion-size, contrast, slice-position, and artifact-severity annotations prevent a controlled separation of class-frequency effects from intrinsic image difficulty. Future work should include class-balanced training and radiologist-guided error stratification. Seventh, although the external bootstrap confidence intervals and repeated-noise variability describe uncertainty in aggregate evaluation metrics, the current deterministic classifier does not provide calibrated per-image predictive uncertainty or separate aleatoric and epistemic components. Future work will incorporate Deep Ensembles, Monte Carlo Dropout, or Evidential Deep Learning and will evaluate calibration and selective prediction using negative log-likelihood, Brier score, expected calibration error, reliability diagrams, and risk–coverage analysis, particularly under external domain shift. Eighth, the layer-wise mask-placement experiment is a controlled single-seed ablation. It provides empirical support for distributing masked reconstruction supervision across the stack but does not establish a formal theoretical guarantee or quantify variability across repeated training runs. Multi-seed evaluation and analysis of feature dependence across blocks are required to characterize the mechanism more fully. Future research will also explore lightweight designs and extend the framework to tasks like segmentation and detection.

## 4. Summary

This study presented S^2^MDAE, which combines layer-wise masked reconstruction, Gaussian corruption, and stacked convolutional encoder–decoder blocks for brain MRI classification. The model achieved 87.207% Accuracy on the primary dataset, and the ablation and representation analyses supported the contribution of its principal components. External evaluation achieved 67.254% Accuracy and 66.009% Macro-F1 without retraining, demonstrating partial cross-dataset transfer but also a substantial domain-shift gap. Future work will focus on multi-center validation, realistic low-SNR data, calibrated uncertainty estimation, and repeated-seed evaluation.

## Figures and Tables

**Table 1:** Architectural comparison of Stacked DAE, MAE, and S^2^MDAE.

Aspect	Stacked DAE	MAE	S^2^MDAE
Corruption location	Corruption is applied to the input or hidden representation of the DAE being trained.	One mask is sampled on image patches before the encoder.	Gaussian corruption is applied to the image input, and an independent mask is sampled at every stacked block.
Pre-training organization	Typically greedy and layer-wise, followed by stacking and supervised fine-tuning.	A single input-level masked reconstruction objective trains the deep encoder and lightweight decoder.	Image-level and feature-level reconstruction losses from all blocks are summed and jointly optimized.
Decoder arrangement	A local decoder is used for each layer during denoising pre-training.	One asymmetric lightweight decoder reconstructs masked image patches.	Each stacked block contains its own decoder and reconstructs the representation entering that block.
Downstream use	The stacked encoders are fine-tuned for the target task.	The encoder is retained and the reconstruction decoder is discarded.	The complete encoder stack is connected to a classifier and fine-tuned end-to-end.

**Table 2:** Class distribution of the primary dataset across the two official partitions. Percentages are calculated within the Training and Testing partitions.

Class	Training Count	Training (%)	Test Count	Test (%)	Total
Glioma	1321	23.13	300	22.88	1621
Meningioma	1339	23.44	306	23.34	1645
No tumor	1595	27.92	405	30.89	2000
Pituitary	1457	25.51	300	22.88	1757
Total	5712	100.00	1311	100.00	7023

**Table 3:** Classification performance under different data settings. Precision, Recall, and F1-score are macro-averaged values.

Setting	Accuracy (%)	Precision (%)	Recall (%)	F1-Score (%)
Proposed setting (Ours)	87.207	86.311	86.230	86.142
Original dataset	90.542	90.212	89.886	90.000
Noisy dataset without preprocessing	85.672	84.195	83.827	83.759

**Table 4:** Ablation and baseline comparison. Precision, Recall, and F1-score are macro-averaged values.

Method	Accuracy (%)	Precision (%)	Recall (%)	F1-Score (%)
S^2^MDAE (full)	87.207	86.311	86.230	86.142
No pre-training	80.778	80.243	79.611	79.271
No masking	77.193	75.710	75.649	75.279
No noise injection	84.134	83.374	83.011	82.801
CNN baseline	64.683	60.921	63.157	61.319
ResNet50	84.897	84.397	83.754	83.819

**Table 5:** Controlled ablation of layer-wise independent masking versus input-layer-only masking. Precision and Recall are macro-averaged. Each row is one run with the same seed and training protocol.

Masking Strategy	Accuracy (%)	Balanced Accuracy (%)	Precision (%)	Recall (%)	Macro-F1 (%)	Weighted-F1 (%)
Layer-wise independent masks	71.777	70.140	69.828	70.140	69.300	70.639
Input-layer-only mask	65.828	65.375	65.127	65.375	64.293	65.390

**Table 6:** Supplementary sensitivity results for alternative pre-training mask ratios. Precision, Recall, and F1-score are macro-averaged values.

Mask Ratio (%)	Accuracy (%)	Precision (%)	Recall (%)	F1-Score (%)
50	68.574	66.491	67.339	65.455
60	71.014	69.330	69.748	69.420
90	69.718	67.902	68.139	67.939

**Table 7:** Performance retention under Gaussian noise. Values for non-zero σ are mean ± standard deviation over five deterministic noise realizations and are normalized to each model’s own σ=0 result.

Model	Metric	σ=0	σ=0.05	σ=0.10	σ=0.20	σ=0.30
Full S^2^MDAE	Accuracy retention (%)	100.00	72.93±0.16	39.08±0.04	37.89±0.00	37.89±0.00
Full S^2^MDAE	Macro-F1 retention (%)	100.00	60.78±0.13	17.56±0.08	14.59±0.00	14.59±0.00
No-noise ablation	Accuracy retention (%)	100.00	63.74±0.12	42.49±0.15	37.33±0.00	37.33±0.00
No-noise ablation	Macro-F1 retention (%)	100.00	43.73±0.17	24.55±0.24	14.45±0.00	14.45±0.00

**Table 8:** Layer-wise linear-probe and class-separability results. Accuracy and Macro-F1 are reported as percentages.

Stage	Block	Dimension	Accuracy (%)	Macro-F1 (%)	Silhouette
Pre-trained	Block 1	64	78.719	77.089	−0.0920
Pre-trained	Block 2	128	72.082	70.585	−0.0859
Pre-trained	Block 3	256	71.167	69.630	−0.1097
Fine-tuned	Block 1	64	77.727	76.144	0.0147
Fine-tuned	Block 2	128	80.931	79.863	0.0217
Fine-tuned	Block 3	256	81.846	80.439	0.0631

**Table 9:** Cross-dataset external evaluation on the duplicate-screened test set of the second public brain MRI dataset. All models were trained only on the primary dataset and evaluated without external retraining or fine-tuning. Precision, Recall, and F1-score are macro-averaged values.

Model	Accuracy (%)	Balanced Acc. (%)	Precision (%)	Recall (%)	Macro-F1 (%)	Weighted-F1 (%)
S^2^MDAE (full)	67.254	67.149	66.289	67.149	66.009	67.002
No pre-training	64.332	64.222	63.066	64.222	62.350	63.194
CNN baseline	44.048	42.009	43.631	42.009	42.056	43.295

## Data Availability

Third-party datasets were analyzed in this study. The primary dataset, referred to in this article as the Brain Tumor MRI Dataset, was obtained from the Baidu AI Studio dataset platform. The dataset is not readily available through a dataset-specific persistent link because such a link could not be identified. The Baidu AI Studio dataset portal is available at https://aistudio.baidu.com/datasetoverview (accessed on 24 June 2026), and access to the original dataset is subject to the platform’s continued availability and access conditions. Further inquiries regarding the primary dataset should be directed to the first author (R.L.). The external evaluation dataset, Brain Tumor MRI Dataset (Glioma, Meningioma, Pituitary, No Tumor), Version 4, is openly available in Mendeley Data at https://doi.org/10.17632/zwr4ntf94j.4. No new patient data were collected or generated in this study. The source code used for model implementation and evaluation is openly available on GitHub at https://github.com/limou66/S2MDAE (accessed on 24 June 2026).
